# Evaluation of maxillary sinus dimensions and volume using cone beam computed tomography in patients with unilaterally displaced palatal and buccal maxillary canines

**DOI:** 10.1007/s11282-022-00663-6

**Published:** 2022-11-15

**Authors:** Elham S. Abu Alhaija, Ahed M. AlWahadni, Akram Al-Tawachi, Saba O. Daher, Hasan O. Daher

**Affiliations:** 1 College of Dental Medicine, QU Health, Qatar University, P.O. Box: 2713 Doha, Qatar; 2 Department of Prosthodontics, Faculty of Dentistry, Jordan University of Science and Technology, Irbid, P.O. Box 3030 Jordan; 3 Private Practice, Dubai, United Arab Emirates; 4 Faculty of Medicine, Jordan University of Science and Technology, Irbid, P.O. Box 3030 Jordan

**Keywords:** Maxillary sinus dimensions, Maxillary sinus volume, Maxillary canine, Palatal displacement, Buccal displacement, Cone Beam computed tomography

## Abstract

**Objectives:**

To evaluate and compare the maxillary sinus (MS) dimensions and volume in unilaterally displaced palatal and buccal maxillary canines.

**Methods:**

CBCT images for 133 patients were included in the study. Maxillary canines were unilaterally displaced palatally in 83 patients (PDCs) and buccally in 50 patients(BDCs). The following variables were measured: canine position in relation to MS walls, MS pneumatization and MS dimensions and volume.

**Results:**

MS was extended to the incisor region in 10% and 13% and to the canine region in 48% and 23% in BDCs and PDCs subjects, respectively. In BDC subjects, maxillary canine crown tip was more laterally (24.23 mm compared to 22.93 mm (*p* < 0.05)) and closer vertically (5.82 mm compared to 9.58 mm (*p* < 0.001)) to the MS, maxillary canine root tip was closer to the MS anterior (0 mm compared to 1.64 mm (*p* < 0.05)) and lateral (19.70 mm compared to 22.02 mm (*p* < 0.001)) walls and the MS volume (11.57mm^3^ compared to 9.09 mm^3^ (*p* < 0.001)) was increased in the displaced side compared to the non-displaced side. In PDC subjects, a significant difference between the displaced and non-displaced sides was detected in the vertical (3.28 mm compared to 5.89 mm (*p* < 0.001)) and lateral (21.63 mm compared to 24.25 mm (*p* < 0.001)) position of maxillary canine to the MS wall, the anterior (− 0.84 mm compared to 1.13 mm (*p* < 0.05)) and lateral (20.48 mm compared to 22.44 mm (*p* < 0.001)) position of canine root tip to the MS and the MS volume (7.71mm^3^ compared to 9.14mm^3^ (*p* < 0.001)). PDC sides differed from BDC sides in the lateral and vertical position of canine crown tip to MS and in MS volume. PDC showed negative association with MS volume and anteroposterior skeletal relationship and a positive association with MS height.

**Conclusions:**

PDCs subjects have a reduced MS volume and BDCs subjects have an increased MS volume. PDCs are associated with reduced MS volume, increased MS height and Class III skeletal relationship.

## Introduction

The maxillary sinus (MS) is the largest of the paranasal sinuses in the facial bone structure which is located within the maxillary body. It is generally pyramidal in shaped [1] with its base located in the lateral nasal wall, and its apex in the zygomatic process of the maxilla. At birth, its volume ranges from 6 to 8 cm^3^, and then, it increases to reach its final size (ranges from 8.6 to 24.9 cm^3^) between 12 and 15 years [2]. The MS floor is formed by the alveolar process of the maxilla and sometimes, the posterior teeth roots are in close relationship to the sinus. Also, canine roots may approximate the inferior wall of the MS especially when they are in an impacted position [3, 4].

The canine tooth is usually erupted at 11 to 13 years of age where its root is completely formed by 13 to 15 years of age. From its starting position between the roots of the first primary molar tooth in the first-year of life, the crown of the permanent maxillary canine lies vertically above the first premolar germ at age of 3 to 4 years. The permanent canine then moves forwards and downwards to rest buccal and mesial to deciduous canine root apex. Coulter and Richardson [5] reported that the normal path of canine eruption extends over 22 mm from age 5 to 15 years. The maxillary permanent canine is a common tooth to deviate from its normal path of eruption and become displaced or impacted [6]. In Caucasians, maxillary canine displacement has been reported to be about 2–3% [7]. More maxillary canines are said to be displaced in palatal position (85%) compared to labial position (15%) [8].

MS size previously has been investigated using conventional two-dimensional (2D) radiographs [9] with limited information in terms of defining a complex three-dimensional anatomic structure. Cone beam computed tomography (CBCT) is a three dimensional (3D) image which is recommended by the American Academy of Oral and Maxillofacial Radiology to detect and diagnose any dental problem associated with palatal canine displacement [10]. Haney et al. [11] concluded that 2D and 3D images of impacted maxillary canines can produce different diagnoses and treatment plans and they recommended the use of CBCT to detect problems related to palatally displaced canines.

The MS development might be affected by maxillary dental development. Wehrbein and Diedrich [12] reported a positive correlation between the amount of sinus expansion after dental extraction and the projection length of roots into the sinus. However, studies to evaluate the relationships between the maxillary canine and the MS are limited [13, 14]. The upper permanent canine tooth germ develops in close proximity to the maxillary sinus, therefore, upper canine displacement and MS pneumatization may have an association. This was investigated by Oz et al. [13] who compared the MS volumetric changes after orthodontic traction of impacted canines to the dental arch using CBCT. They reported a significant increase in the MS volume with the orthodontic traction of the impacted canines which were closer to the MS.

Taking into consideration MS anatomical variability and its close position to the erupting maxillary canines, the assessment of MS during orthodontic treatment planning for patients with maxillary canine displacement may be of help to understand the etiology of canine displacement and improve treatment outcome. The null hypothesis was that there would be similar MS volume and dimensions in displaced (BDCs and PDCs) and non-displaced canine groups. The objectives of this study were: -

- To evaluate and compare the MS dimensions (length, height, width) and volume in unilaterally displaced palatal and buccal maxillary canines.
- To evaluate and compare the presence of MS septa and MS pneumatization in unilaterally displaced palatal and buccal maxillary canines.
- To investigate the association of maxillary canine displacements with MS dimensions, MS volume and skeletal patterns (antero-posterior and vertical).

## Material and Methods

Study design: Case control cross-sectional study.

This study was reviewed and approved by the Research Ethical Committee (IRB)/Jordan University of Science and Technology (JUST). The sample of this study was collected over a period of 7 years by three means: database search (existing CBCT that were taken previously for diagnostic purposes as part of comprehensive orthodontic treatment), patients attending orthodontic clinics at the postgraduate dental clinics/Jordan University of Science and Technology (JUST) and referrals by fellow dentists and orthodontists. CBCT images were taken at the Dental Teaching Clinics (DTC)/Jordan University of Science and Technology (JUST). The CBCT images taken between January 2014 and January 2021 were screened for the presence of unilaterally displaced maxillary canines. Inclusion criteria were age ≥ 16 years, no reported breathing problems, non-syndromic and non-cleft patients, with no previous orthodontic treatment, no history of trauma; no root canal treatment and no presence of cysts or other pathologies.

Sample size was calculated using the G*power 3.1.9 program. Based on a study conducted by Oksayan et al. [15] to compare sinus volume in patients with different vertical growth patterns using CBCT (12.41 (4.58) and 13.85 (4.92) in high and normal vertical angle subjects, respectively), a small effect size difference (0.3) was assumed between groups. A total sample size estimate of 97 canines (49 canines/group) at a conventional alpha level (0.05) and desired power (1–β) of 0.90 was calculated.

**Fig. 1:**
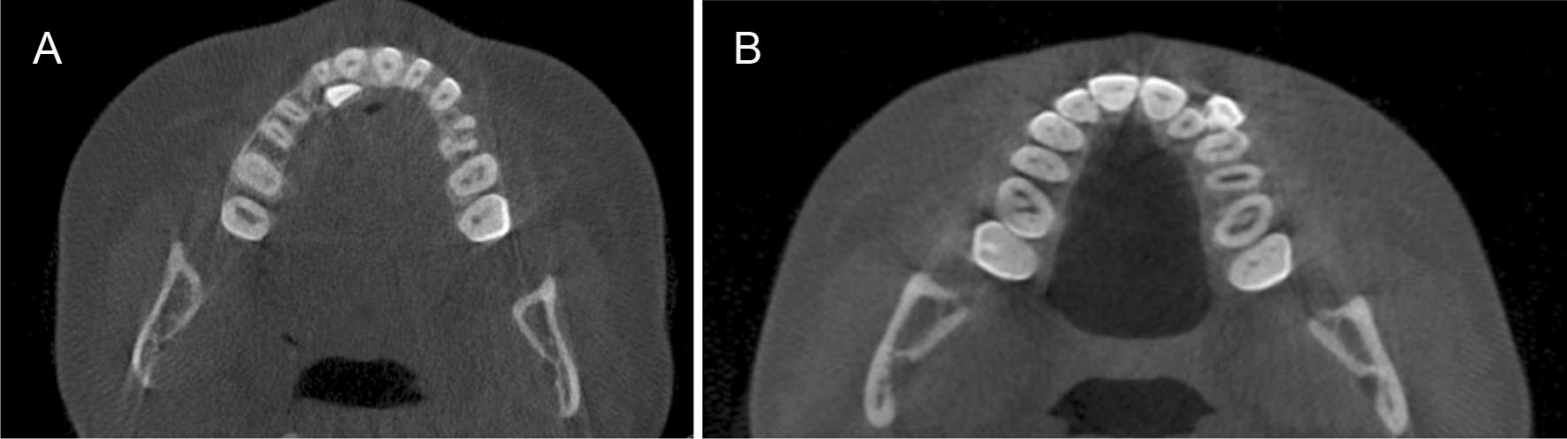
A: Palatally displaced canine (PDC): canines appearing palatal to a line connecting the roots of adjacent teeth at any level of canine crown. B: Buccally displaced canine (BDC): canines appearing buccal to a line connecting the roots of adjacent teeth at any level of canine crown

Canines were determined to be displaced palatally, buccally or not displaced as follows (Fig. 1a, b):

- Palatally displaced canines (PDCs): canines appearing palatal to a line connecting the roots of adjacent teeth at any level of canine crown (*n* = 83).
- Buccal displaced canines (BDCs): canines appearing buccal to a line connecting the roots of adjacent teeth at any level of canine crown (*n* = 50).
- Non displaced canines (NDCs): canines that are normally erupted as should in the dental arch (*n* = 133)

The patient’s gender and age at the time of CBCT imaging were noted. A total of 133 patients (101 females and 32 males; 67 on the right side and 66 on the left side) were included in the study. Age ranged from 16 to 26 years (averaged 18.75 ± 1.57 years). Maxillary canines were unilaterally displaced palatally in 83 patients (63 females and 20 males; 37 on the right side and 46 on the left side, age averaged 18.92 ± 1.73 years) and buccally in 50 patients (38 females and 12 males; 30 on the right side and 20 on the left side, age averaged 18.48 ± 1.23 years).

Of the total PDC subjects, 29 had Class I (ANB° averaged 2.57 ± 0.47), 30 had Class II (ANB° averaged 4.99 ± 0.30), and 24 had Class III (ANB° averaged -0.90 ± 1.00) skeletal malocclusion and 16 subjects had reduced (19.52 ± 1.77), 39 subjects had normal (28.25 ± 2.03) and 28 subjects had increased (34.24 ± 1.83) Maxillary/Mandibular (Max/Mand°) angle.

Within BDC subjects, 17 had Class I (ANB° averaged 2.43 ± 0.47), 29 Class II (ANB° averaged 5.00 ± 0.37) and 4 subjects had Class III (ANB° averaged − 1.25 ± 0.50) skeletal malocclusion. Vertically, 8 subjects had reduced (19.97 ± 1.32), 24 had normal (28.51 ± 1.44), and 18 subjects had increased (34.97 ± 2.02) Max/Mand° angle.

Class I skeletal relationship was considered when ANB angle = 3° ± 1, Class II skeletal relationship was considered when ANB angle was > 4°, and Class III skeletal relationship was considered when ANB angle was < 2°. Vertically, normal Max/Mand relationship was considered when Max/Mand angle = 27° ± 5, reduced Max/Mand angle was considered when Max/Mand angle was < 22°, and increased Max/Mand angle was considered when Max/Mand angle was > 32°.

### CBCT images

A CS 9500 Cone Beam 3D System (Carestream Health, Ro-chester, NY, USA) with a flat panel detector located at the DTC/JUST was the only CBCT apparatus used. The CBCT images were 0.2 mm slices of a medium field of view (FOV), where the maxillofacial area was examined at a tube voltage of 90 kV, a tube current of 10 mA, and an exposure time of 8.01 s. The imaging area was a cylinder with a height of 15 cm (cm) and a diameter of 9 cm, providing 0.2 mm cubic voxels. To ensure that the voltage, current, resolution, threshold, contrast, brightness, field of view (FOV) and patient position did not affect the measurements obtained from the CBCT images, CBCT parameters and patient position were identical in all CBCT scans.

Examinations were performed by 360° rotation with a patient in an upright position and Frankfort Horizontal plane parallel to the ground. CBCT orthogonal views were used for linear measurements. The right and left sinuses were separated and measured individually.

3D reconstruction view was used to aid in assessing the MS.” View control” was changed to view the MS clearly separated from adjacent dental and bony structure by choosing the proper threshold. MS was separated from adjacent structures using volume render tool on all the different views i.e., coronal, axial and sagittal. The outline of the MS was traced following the inner bony surface. Eventually, “Volume measurement” option was used to calculate the total volume of MS volume.

The DICOM files were imported into InVivo software Dental version 6.0.5 (Anatomage, San Jose, Calif) software program for secondary reconstruction and further investigation. CBCT images were evaluated throughout a period of 2 months, by one examiner (A.A.). All images were evaluated in dimmed light using a screen with 1920X1200 pixels.When necessary, the window settings were adjusted to optimize the images for evaluation, and zoomed in as much as needed for a careful evaluation. Images were oriented in three spatial planes. The volumetric accuracy of CBCT scanner was evaluated by measuring the volume of 2 teeth planned for extraction from CBCT using Invivo dental software and later after extraction.

Definitions of the variables included in the study and methods used to measure them are shown in Table 1. All measurements were carried out by one single investigator (A. A.).

A random sample of 13 CBCT images (10% of total sample) where re-evaluated after 2 weeks interval. All measurements were repeated by the same examiner in the same conditions to test intra-examiner reliability. Kappa test was used for the categorical data and dahlberg error for the double measurement was used to calculate the standard error of the method. The average kappa value for the measured categorical variables was 0.96. Dahlberg error ranged from 0.05 mm for MS length to 0.012 mm for the vertical distance from the canine tip to MS (mm) and was 0.04mm3 for MS volume.

### Statistical Analysis

Statistical analysis was performed with the use of the Statistical Package for Social Science (SPSS) computer software (SPSS 23, SPSS Inc., NY, USA). Descriptive statistics were calculated for all the measured variables for each group. The Shapiro–Wilk test was applied to assess normality of numeric data and the result indicated that data were not normally distributed. The Wilcoxon signed-rank test was applied to detect differences between the right and left sides within each group. Differences between the two displacement groups were assessed using Chi-square and Mann Whitney U test for categorical and nominal variables, respectively. Odds ratio (OR) was presented using the binary logistic regression analysis to determine the association of maxillary canine displacement (dichotomized to either BDCs or PDCs) with dimensions and volume of MS and type of skeletal malocclusion. The level of significance was set at P ≤ 0.05.

## Results

Gender differences were not detected within each study group (P > 0.05). Therefore, males and females’ data were pooled together during further analysis. Distribution of displaced and nondisplaced canines in the 2 studied groups in respect to presence and location of MS septa, anterior and inferior sinus extension in relation to canines, Pearson chi-square and P values are shown in Table 1.

**Table 1:** Definitions of the variables used in the study

	Variable	Definition
Canine displacement (Fig. 1a, b)	Palatally displaced canine Figure 1a	Canines appearing palatal to a line connecting the roots of adjacent teeth at any level of canine crown. Coronal plane set at the posterior margin of hard palate and sagittal plane in the midline of the hard palate
	Buccally displaced canine Figure 1b	Canines appearing buccal to a line connecting the roots of adjacent teeth at any level of canine crown. Coronal plane set at the posterior margin of hard palate and sagittal plane in the midline of the hard palate
Maxillary canine to maxillary sinus Figures 2, 3, 4	Canine crown tip and root apex anteriorly (mm) Figure 2	Sagittal view with soft tissue and teeth view-control and full-half sagittal clipping were chosen to do these measurements as follows: -The distance between maxillary canine crown tip (CCT) and root apex (CRA) to the most anterior point of maxillary sinus (AWMS). If sinus is extended anterior to canine tip, a minus sign was given
	Canine crown tip and root apex laterally (mm) Figure 3	Frontal view with soft tissue and teeth view-control and full-half coronal clipping were chosen to do these measurements as follows: -The distance between maxillary canine crown tip (CCT) and root apex (CRA) to the most lateral point of maxillary sinus (LWMS)
	Canine crown tip and root apex inferior (mm) Figure 4	Frontal view with soft tissue and teeth view-control and full-half coronal clipping were chosen to do these measurements as follows: -The distance between maxillary canine crown tip (CCT) and root apex (CRA) to the most inferior point of maxillary sinus (IWMS). If sinus is extended inferior to canine tip, a minus sign was given
Sinus volume (mm^3^) (Fig. 5)	Sinus volume (mm^3^) (Fig. 5)	Frontal view with soft tissue and teeth view-control and full-half coronal clipping were chosen initially. The outline of the maxillary sinus was traced and isolated following the inner bony surface using the free hand sculpture. Any other structures showing when MS is viewed from different sections were trimmed. Maxillary sinus volume was calculated by InViVo dental Anatomage software
Maxillary sinus dimensions (Figs. 6–8)	Maxillary sinus width (mm) (Fig. 6)	Frontal view with soft tissue1 view-control and full-half coronal clipping were chosen. The distance between the most medial and the most lateral point of maxillary sinus viewed on coronal section was measured
	Maxillary sinus height (mm) (Fig. 7)	Frontal view with soft tissue1 view-control and full-half coronal clipping were chosen. The distance between the most superior and the most inferior point of maxillary sinus viewed on coronal section was measured
	Maxillary sinus length (mm) (Fig. 8)	Sagittal view with soft tissue1 view-control and full-half sagittal clipping were chosen. The distance between the most anterior and the most posterior point of maxillary sinus viewed on sagittal section was measured
Maxillary sinus septa	Presence of maxillary sinus septa (Fig. 9)	The presence of maxillary sinus septa was determined on the axial sections of CBCT images for each maxillary sinus. Septa were identified as available in cases with > 4 mm septa height on the sagittal sections
Maxillary sinus extension	Anterior extension	The most anterior limit on maxillary sinus viewed on sagittal section. It was recorded in respect to upper teeth (first premolar, second premolar or canine areas)

**Fig. 2:**
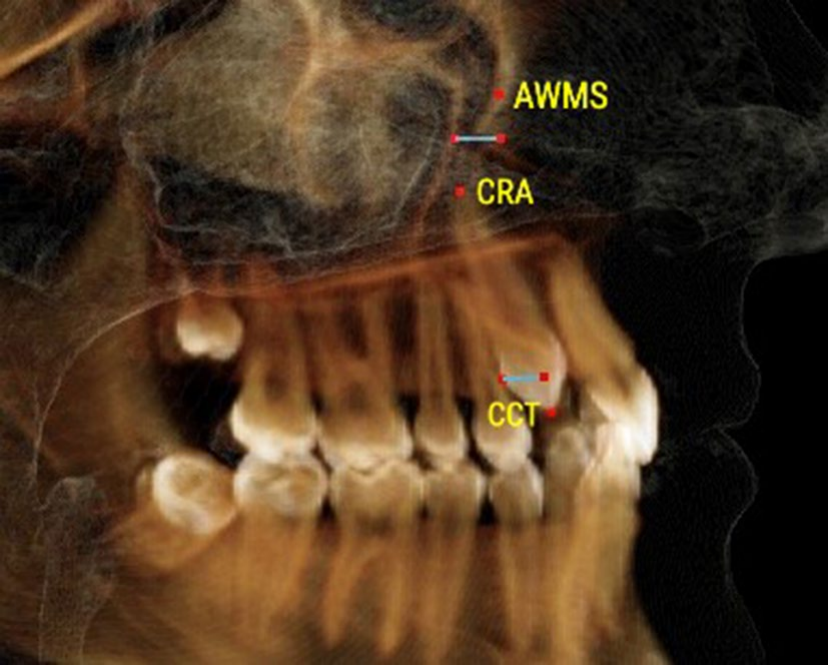
The distance between maxillary canine crown tip (CCT) and root apex (CRA) to the most anterior point of maxillary sinus (AWMS) using the sagittal view with soft tissue and teeth view-control and full-half sagittal clipping

**Fig. 3:**
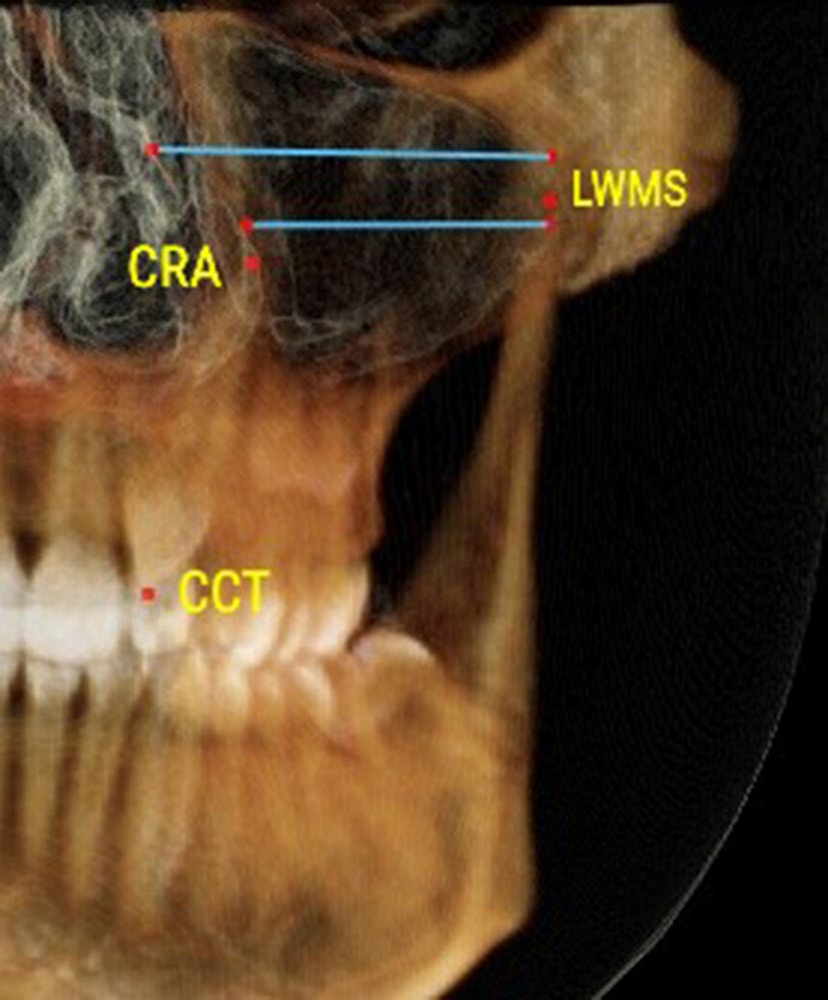
The distance between maxillary canine crown tip (CCT) and root apex (CRA) to the most lateral point of maxillary sinus (LWMS) using frontal view with soft tissue and teeth view-control and full-half coronal clipping

**Fig. 4:**
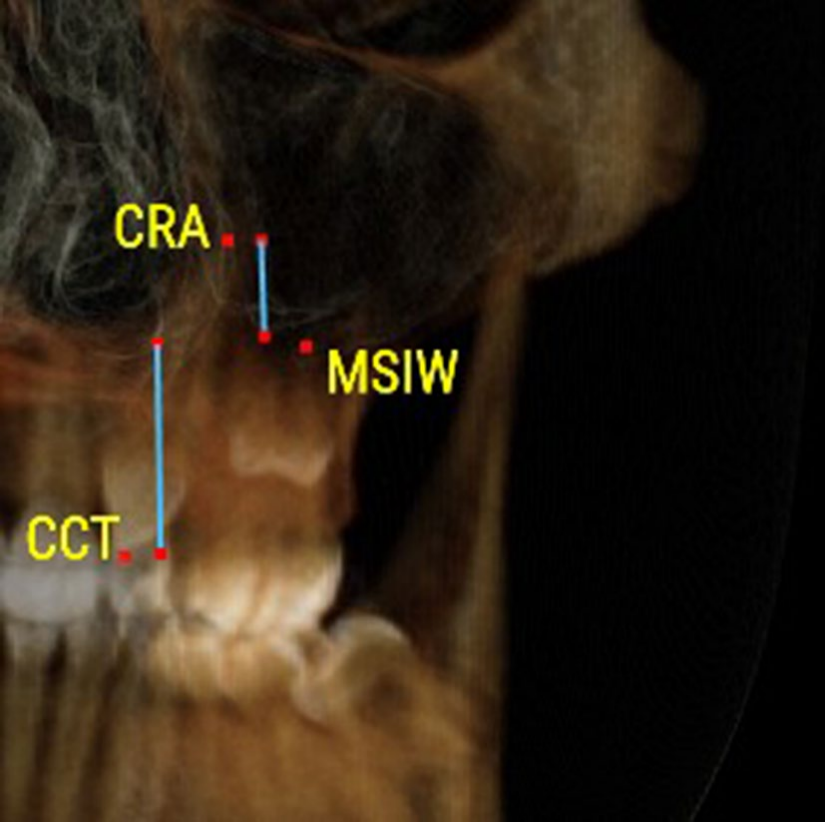
The distance between maxillary canine crown tip (CCT) and root apex (CRA) to the most inferior point of maxillary sinus (IWMS) using frontal view with soft tissue and teeth view-control and full-half coronal clipping

**Fig. 5:**
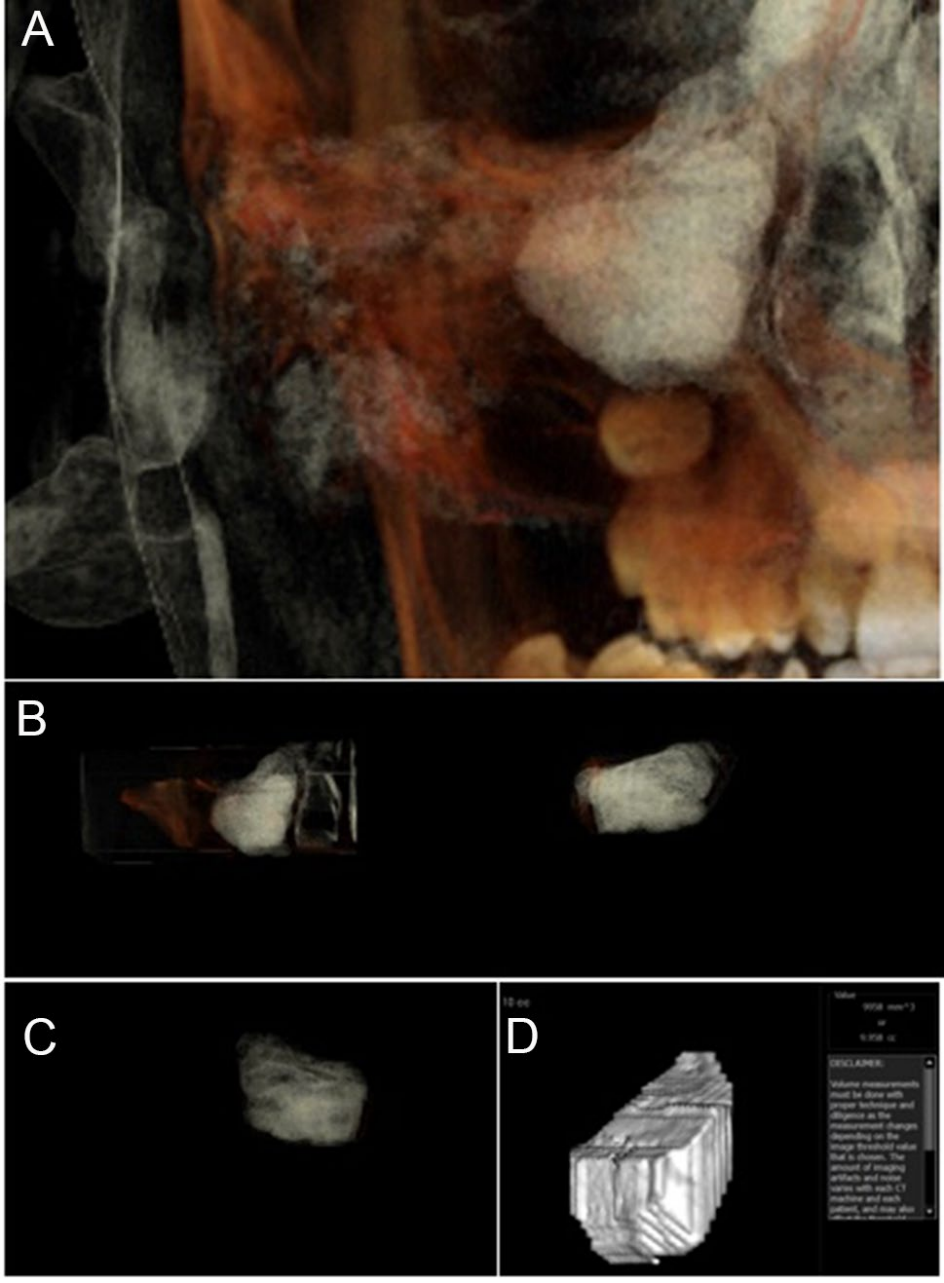
A-Frontal view with soft tissue and teeth view-control and full-half coronal clipping were chosen initially. B-C The outline of the maxillary sinus was traced and isolated following the inner bony surface using the free hand sculpture. Any other structures showing when MS is viewed from different sections were trimmed. D- Maxillary sinus volume calculation using the volume option

**Fig. 6:**
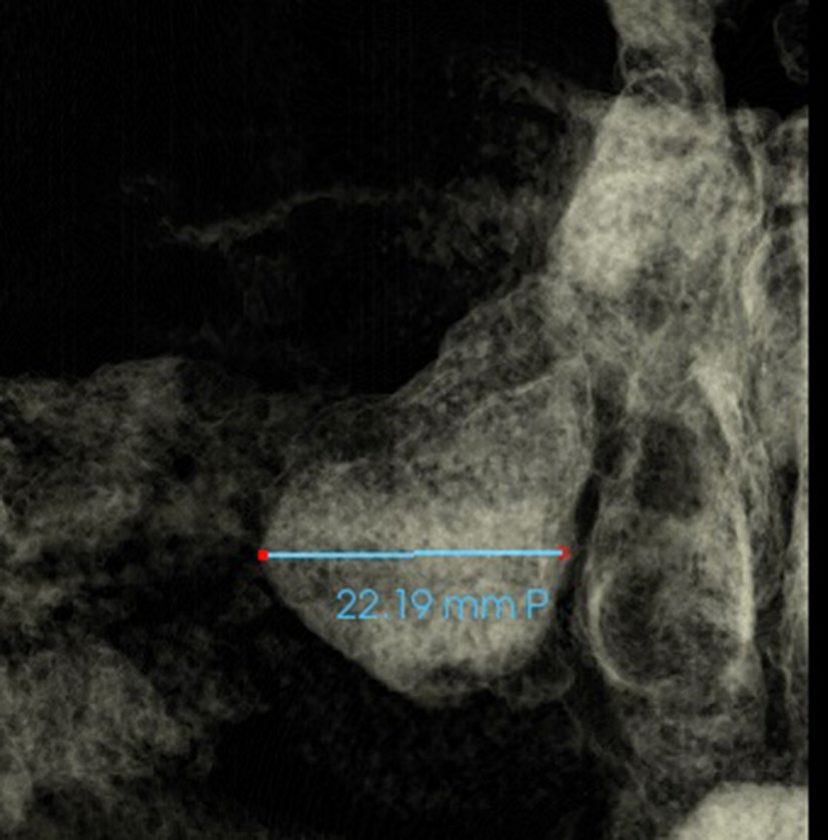
Maxillary sinus width measured as the distance between the most medial and the most lateral point of maxillary sinus viewed on coronal section (Frontal view with soft tissue1 view-control and full-half coronal clipping)

**Fig. 7:**
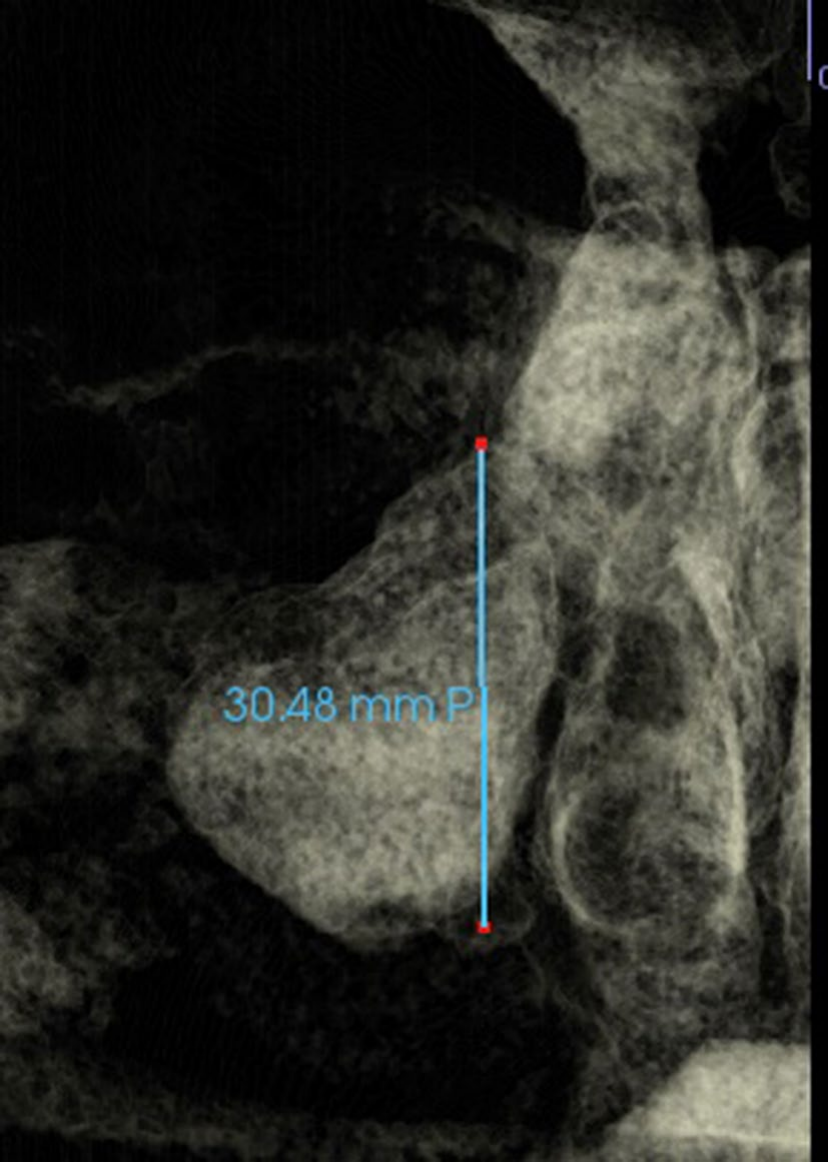
Maxillary sinus height measured as the distance between the most superior and the most inferior point of maxillary sinus viewed on coronal section (Frontal view with soft tissue1 view-control and full-half coronal clipping)

**Fig. 8:**
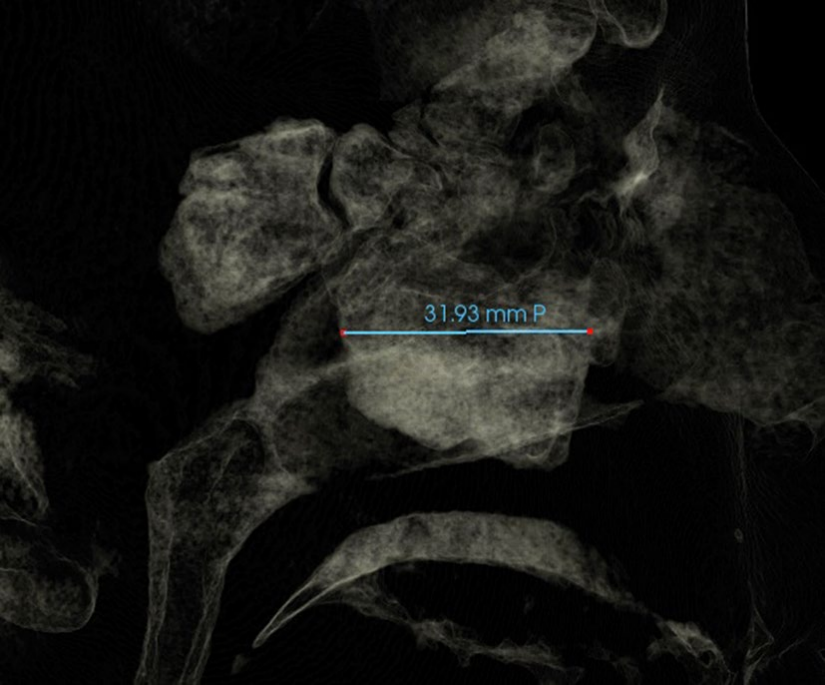
Maxillary sinus length measured as the distance between the most anterior and the most posterior point of maxillary sinus viewed on sagittal section (Sagittal view with soft tissue1 view-control and full-half sagittal clipping)

**Fig. 9:**
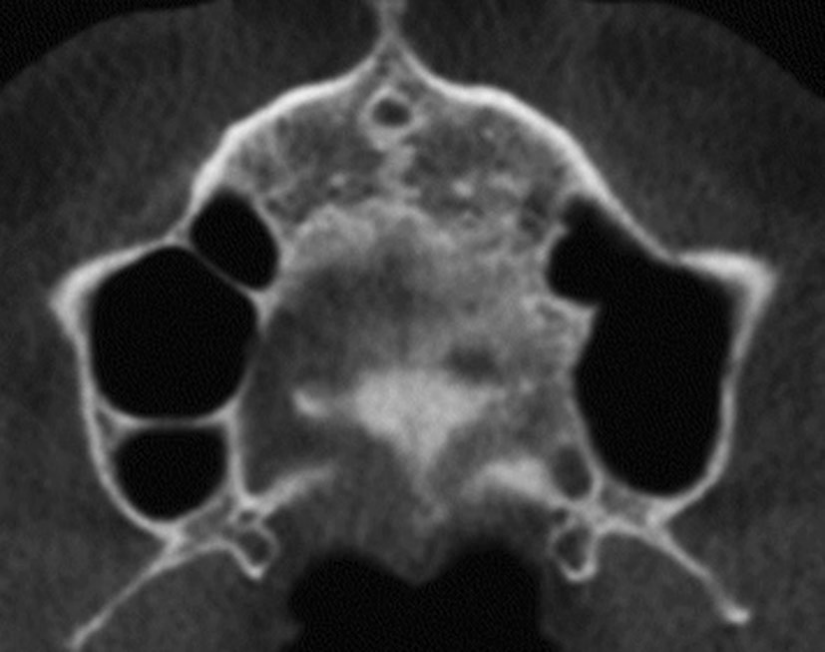
Presence of maxillary sinus septa

There was a statistically significant association (moderate association, φ = 0.217) between the presence/location of septa on the non-displaced side and the type of canine displacement, χ2 (df) = 6.26 (2), *p* = 0.044. MS septa were detected in 8% and 6% of BDCs’ and PDCs’ non-displaced sides, respectively. On the displaced side, MS septa were present in 6% and 11% of BDC and PDC subjects, respectively (χ2 (df) = 5.37 (2), *p* = 0.068).

There was a statistically significant association (strong association, φ = 0.302) between the presence of anterior pneumatization of MS on the displaced side and the type of canine displacement χ2 (df) = 12.16 (4), *p* = 0.016. More MS anterior pneumatization was detected in the maxillary displaced canines’ sides. MS was extended to the incisor region in 10% and 13% and to the canine region in 48% and 23% in BDC and PDC subjects, respectively. On the non-displaced canine side, MS was extended to the incisor region in 10% and 6%, to the canine region in 34% and 36% in BDC and PDC subjects, respectively (P > 0.05).

Means, standard deviations (SDs) of the studied variables for the right and left sides of the unilaterally displaced canine (BDC and PDC) subjects, Wilcoxon signed mean ranks, standardized test statistics and P values in the studied groups are shown in Table 2.

**Table 2:** Distribution of displaced and non-displaced maxillary canine subjects according to presence and location of maxillary septa and anterior extension of maxillary sinus, chi-square coefficients (R), degree of freedom (df), Cramer’s *V* and *p* values

		BDCs subjects	PDCs subjects	Chi-Square R (df)	Cramer’s V	*p* value
*Non-displaced displaced side*						
Location of septa	No septa	46 (92%)	78 (94%)	6.26 (2)	0.023	0.044*
	Anterior	4 (8%)	1 (1%)			
	Posterior	0 (0%)	4 (5%)			
Anterior pneumatization	Central incisor	0 (0%)	3 (4%)	9.66 (5)	0.270	0.085NS
	Lateral Incisor	5 (10%)	2 (2%)			
	Canine	17 (34%)	30 (36%)			
	First premolar	25 (50%)	34 (41%)			
	Second premolar	3 (6%)	9 (11%)			
	First molar	0 (0%)	5 (6%)			
*Displaced side*						
Location of septa	No septa	47 (94%)	74 (89%)	5.37 (2)	0.201	0.068NS
	Anterior	0 (0%)	7 (8.5%)			
	Posterior	3 (6%)	2 (2.5%)			
Anterior pneumatization	Lateral Incisor	5 (10%)	11 (13%)	12.16 (4)	0.302	0.016*
	Canine	24 (48%)	19 (23%)			
	First premolar	14 (28%)	40 (48%)			
	Second premolar	7 (14%)	9 (11%)			
	First molar	0 (0%)	4 (5%)			

*NS* not significant

*Significant at *p* < 0.05

In BDC subjects, significant differences between the displaced and non-displaced sides were detected. In the displaced side, maxillary canine crown tip was more laterally (*p* < 0.05) and closer vertically to the MS (*p* < 0.001), maxillary canine root tip was closer to the MS anterior (*p* < 0.05) and lateral (*p* < 0.001) walls and the MS volume was increased (*p* < 0.001) compared to the non-displaced side. While in the displaced side of PDC subjects, maxillary canine crown tip was closer to the MS wall laterally (*p* < 0.001) and vertically (*p* < 0.001), maxillary canine root tip was closer to the MS anterior (*p* < 0.05) and lateral (*p* < 0.001) walls and the MS volume was reduced (*p* < 0.05) compared to the non-displaced side.

The Mann–Whitney U standardized test statistics of the displaced and the non-displaced sides in the 2 studied groups are shown in Table 3. Between the displaced canine sides, PDC subjects had their maxillary canine crown tip closer to MS wall laterally (*p* < 0.001) and vertically (*p* < 0.01) and a reduced MS volume (*p* < 0.001) as compared to the BDC subjects.

**Table 3:** Means, standard deviations (SD) and medians of the maxillary sinus variables, Wilcoxon signed rank standardized test statistics and significance (*p*-value) between displaced and non-displaced sides of maxillary unilateral displaced canine (BDC and PDC) subjects

Variable	Displaced side Mean (SD) Median		Non-displaced side Mean (SD) Median		Standardized test statistics	*p*-value
BDCs (*n* = 50 subjects)						
Anterior distance canine tip-MS (mm)	3.39 (7.26)	5.41	3.30 (6.26)	4.47	− 0.46	0.647NS
Lateral distance canine tip-MS (mm)	24.46 (3.67)	24.23	22.77 (2.94)	22.93	− 2.33	0.020*
Vertical distance canine tip-MS (mm)	6.69 (5.30)	5.82	9.87 (6.72)	9.68	− 3.41	< 0.001***
Anterior distance canine root tip-MS (mm)	− 0.19 (3.77)	0.00	1.63 (3.13)	1.64	− 2.48	0.013*
Lateral distance canine root tip-MS (mm)	19.34 (4.97)	19.70	22.10 (2.72)	22.02	− 3.48	< 0.001***
Vertical distance canine root tip-MS (mm)	− 0.71 (5.65)	− 0.62	− 0.65 (4.09)	− 1.61	− 0.09	0.925NS
MS volume (cm^3^)	12.73 (5.32)	11.57	10.31 (4.34)	9.09	− 3.82	< 0.001***
MS length (mm)	32.49 (4.36)	32.83	32.16 (3.98)	32.18	− 0.34	0.732NS
MS width (mm)	24.70 (5.56)	24.58	24.76 (4.85)	24.11	− 0.14	0.889NS
MS height (mm)	28.69 (6.24)	28.01	28.33 (5.72)	27.81	− 0.13	0.896NS
Vertical distance first molar root-MS (mm)	0.41 (2.28)	0.17	0.71 (2.02)	0.44	− 0.83	0.409NS
*PDCs (n = 83 subjects)*						
Anterior distance canine tip-MS (mm)	2.04 (5.91)	2.64	0.79 (5.85)	1.78	− 1.55	0.122NS
Lateral distance canine tip-MS (mm)	22.16 (4.80)	21.63	24.59 (3.73)	24.25	− 3.59	< 0.001***
Vertical distance canine tip-MS (mm)	4.19 (4.48)	3.28	7.14 (6.23)	5.89	− 3.99	< 0.001***
Anterior distance canine root tip-MS (mm)	− 0.66 (4.92)	− 0.84	0.80 (4.26)	1.13	− 2.22	0.027*
Lateral distance canine root tip-MS (mm)	20.16 (4.74)	20.48	22.41 (2.78)	22.44	− 3.97	< 0.001***
Vertical distance canine root tip-MS (mm)	− 2.10 (5.73)	− 2.13	− 0.69 (4.21)	0.00	− 1.64	0.100NS
MS volume (cm^3^)	8.30 (2.50)	7.71	9.20 (2.79)	9.14	− 2.55	0.011*
MS length (mm)	32.43 (4.07)	32.57	32.82 (4.53)	33.36	− 1.41	0.159NS
MS width (mm)	25.17 (4.98)	24.35	25.66 (5.01)	25.39	− 1.23	0.219NS
MS height (mm)	29.40 (5.21)	29.63	29.34 (4.77)	29.57	− 0.24	0.810NS
Vertical distance first molar root-MS (mm)	0.44 (1.93)	0.80	0.56 (1.78)	0.00	− 0.10	0.918NS

*NS* non-significant, *MS* Maxillary Sinus

*p < 0.05, ***p < 0.001

The non-displaced sides of PDC and BDC subjects were similar except for the position of maxillary canine crown tip in relation to the MS walls; anteriorly, laterally and vertically (*p* < 0.05); MS was closer to PDC’s crown tip anteriorly and vertically and to BDC’s crown tip laterally.

Table 4 shows the output of the binary logistic regression analysis. Three predictor variables emerged to be significant for predicting odds of PDC happening; MS volume (*p* < 0.001), MS height (*p* < 0.001) and A-P skeletal pattern (*p* = 0.014). The odds of developing PDC increase by 1.7 times as the MS volume decreases by 1 unit. Also, as MS height increases by 1 unit, the odds of developing PDC increase by 22%. Regarding the A-P skeletal pattern, if the subject has Class II skeletal relationship, then the odds of developing PDC decrease by 91% compared to Class III skeletal pattern. In other words, if the subject has Class III skeletal pattern, then the odds of developing PDC increases by 11times compared to Class II subjects (Table 5).

**Table 4:** Medians of the maxillary sinus variables in the displaced and non-displaced sides of PDCs’ and BDCs’ subjects, the Mann–Whitney U standardized test statistics and *p*-value

Variable	BDC Median	PDC Median	Mean Whitney U	Standardized Test statistics	*p*-value
*Displaced side*					
Anterior distance canine tip-MS (mm)	5.41	2.64	1684.00	− 1.82	0.07NS
Lateral distance canine tip-MS (mm)	24.23	21.63	1397.00	− 3.15	0.002**
Vertical distance canine tip-MS (mm)	5.82	3.28	1512.00	− 2.61	0.009**
Anterior distance canine root tip-MS (mm)	0.00	− 0.84	1918.00	− 0.73	0.465NS
Lateral distance canine root tip-MS (mm)	19.70	20.48	1875.00	− 0.93	0.354NS
Vertical distance canine root tip-MS (mm)	− 0.62	− 2.13	1786.00	− 1.34	0.179NS
MS volume (cm^3^)	11.57	7.71	1061.00	− 4.71	*p* < 0.001***
MS length (mm)	32.83	32.57	2037.00	− 0.18	0.860NS
MS width (mm)	24.58	24.35	1993.00	− 0.38	0.705NS
MS height (mm)	28.01	29.63	1930.00	− 0.67	0.502NS
Vertical distance first molar root-MS (mm)	0.17	0.80	2056.00	− 0.09	0.931NS
*Non-displaced side*					
Anterior distance canine tip-MS (mm)	4.47	1.78	1574.00	− 2.33	0.020*
Lateral distance canine tip-MS (mm)	22.93	24.25	1451.00	− 2.90	0.004**
Vertical distance canine tip-MS (mm)	9.68	5.89	1529.50	− 2.54	0.011*
Anterior distance canine root tip-MS (mm)	1.64	1.13	1805.50	− 1.23	0.210NS
Lateral distance canine root tip-MS (mm)	22.02	22.44	1918.00	− 0.73	0.466NS
Vertical distance canine root tip-MS (mm)	− 1.61	0.00	1978.00	− 0.45	0.652NS
MS volume (cm^3^)	9.09	9.14	1922.50	− 0.71	0.479NS
MS length (mm)	32.18	33.36	1826.00	− 1.16	0.247NS
MS width (mm)	24.11	25.39	1858.00	− 1.01	0.313NS
MS height (mm)	27.81	29.57	1807.00	− 1.24	0.214NS
Vertical distance first molar root-MS (mm)	0.44	0.00	1917.00	− 0.74	0.459NS

*NS* non-significant, *MS* Maxillary Sinus

*p < 0.05, **p < 0.001, ***p < 0.001

**Table 5:** Binary regression output, odd ratios (OR), and 95 *per cent* confidence intervals (95% C.I.) to predict the odds of BDCs (coded as 1) PDCs (coded as 3) in respect to maxillary sinus dimensions, volume and skeletal relationship

Variable	B	Standard error	Wald	Degree of freedom	Significance	Odd ratio (OR)	95% C.I. for OR
MS volume (cm^3^)	0.53	0.10	27.31	1	< 0.001***	0.59	0.48–0.72
MS length (mm)	0.09	0.07	1.71	1	0.191NS	1.09	0.96–1.25
MS width (mm)	0.10	0.06	3.01	1	0.083NS	1.11	0.99–1.24
MS height (mm)	0.20	0.06	11.54	1	< 0.001***	1.22	1.09–1.37
Vertical distance first molar root-MS (mm)	− 0.06	0.15	0.17	1	0.677NS	0.94	0.71–1.25
Anterior pneumatization	0.38	0.29	1.77	1	0.183NS	1.47	0.84–2.57
A-P Skeletal pattern (Coded as 1 = Class 1 and 2 = Class 2 and 3 = Class 3)							
A-P Skeletal pattern (Class 1 as reference)			8.84	2	0.012*		
A-P Pattern (Class II)	− 0.98	0.63	2.41	1	0.120NS	0.38	0.11–1.29
A-P Pattern (Class III)	1.48	0.91	2.61	1	0.106NS	4.38	0.73–26.18
A-P Skeletal pattern (Class 3 as reference)							
A-P Pattern (Class II)	− 2.45	0.86	8.20	1	0.004**	0.09	0.02–0.46
Vertical Skeletal pattern (Coded as 0 = normal, 1 = short, and 3 = long face)							
Vertical Skeletal pattern (Normal face as reference)			1.59	2	0.452NS		
Vertical pattern (Short face)	0.41	0.64	0.41	1	0.524NS	1.51	0.43–5.33
Vertical pattern (Long face)	− 0.56	0.66	0.72	1	0.398NS	0.57	0.16–2.08
Vertical Skeletal pattern (Long face as reference)							
Vertical pattern (Short face)	0.97	0.77	1.58	1	0.209NS	2.63	0.58–11.91
Overall significance of the model, *p* = 0.005 Hosmer and Lemeshow test, *p* = 0.087) Cox and Snell R square = 0.404							

*NS* not significant, *MS* Maxillary sinus, *A-P* Antero-posterior

*p < 0.05, **p < 0.01, ***p < 0.001

## Discussion

Although the relationship between the MS volume and shape and upper posterior teeth have been investigated before [16, 17], scarce information is available regarding the association between the maxillary canine displacements and MS dimension and volume. Hence, the present investigation was undertaken to determine the association of the PDC and BDC with the MS dimension and volume in unilaterally displaced maxillary canines. Only, subjects with unilateral canine displacement were included to avoid any confounding factors.

The MS usually present at birth and with age and increases in size afterwards. The most extensive period of growth occurs during the first 8 years and, by the end of the 16th year, the maximal values of all diameters and volume usually are reached [18]. The age of included subjects in this study was at least 16 years to allow for the MSs to reach its adult size and the maxillary canines to fully erupt into its final position.

The vast majority of previous studies demonstrated that the dimensions of the maxillary sinuses were larger in males than in females [19] which was explained by male’s higher functional need (due to bigger body size and larger craniofacial skeleton). However, in the current study, males and females showed insignificant differences in MS dimensions and volume. This was in partial agreement with Urooge and Patil [20] who reported a statistically insignificant gender difference with respect to the maxillary sinus length, height, area and volume. It also is possible that the small number of males in the current study may have masked any gender differences.

In the current study, CBCT images were used to measure the maxillary sinus dimensions and volume. These images were taken for patients to detect anomalies associated with displaced canines which was in line with the recommendations of the American Academy of Oral and Maxillofacial Radiology [10]. Bjerklin and Ericsson [21] reported that treatment plans of 43.7% of their cases were altered on basis of the additional information gained from the CBCT investigation. Maxillary sinus height, width, length and volume and the 3D measurements of maxillary canine crowns and roots are not feasible with periapical or panoramic radiographs.

In the present study, MS anterior pneumatization into the canine area was more pronounced in BDC subjects (48% of the displaced side and 34% on non-displaced side) whereas in PDC subjects, it was found in 23% of the displaced side and in 36% of the non-displaced side. This finding is comparable to that reported by Kopecka et al. [22] but lower than that (69%) reported by others [14, 23]. Additionally, MS pneumatization in the incisor region was found in 10% and 13% of buccal and palatal canine displacement sides, and in 10% and 6% of canine non-displacement sides, respectively. This was lower than 15.5% reported by Zhang et al. [23] However, this was in agreement with Kopecka et al. [22] who suggested that the more MS pneumatization to the canine area occurred in a type I vertical relationship between MS and canine apex (canine apices located at more than 2 mm distance below the sinus floor) followed by type II (less than 2 mm distance) and type III relationship (interlock). MS pneumatization is related to dental position and the variation between displaced and nondisplaced sides and between PDC and BDC sides may be explained by the position of the displaced canine which may have prevented or allowed anterior sinus pneumatization. This was in agreement with Oz et al. [12] who reported an increase in MS dimensions after orthodontic traction if the impacted canines were closer with respect to the MS.

When the displaced and non-displaced sides of BDC and PDC subjects were compared, the position of maxillary canine in relation to MS wall showed significant differences which is expected based on the position of the displaced canine. The variation in the vertical, lateral and A-P position of the canines between right and left sides resulted in significant differences between the displaced and non-displaced canines’ sides.

In BDC subjects, MS volume was larger on the displaced side compared to the non-displaced side. This was accompanied by similar MS dimensions (length, height and width) between the displaced and non-displaced sides. The lack of correlation between MS dimensions and MS volume may be attributed to the method used for their measurement. In the current study, the MS length, width and height were measured as a line between the most prominent points on MS walls at a specific location which does not reflect the actual dimension throughout the MS wall.

In the current study, although the BDC crown tip was located more laterally on the displaced side, the MS width was similar to the non-displaced side. This may be explained by the closer position of the BDC’s root tip to the MS. This was in agreement with previous reports that the shape and size of the maxillary sinuses were affected by the proximity of the roots [17].

In PDC subjects, the MS volume was reduced and the maxillary displaced canine crown and root tip were closer to the MS laterally as compared to the non-displaced side. Similar to that found in BDC subjects, MS dimensions between displaced and non-displaced sides did not differ significantly. The lack of correlation between MS dimensions and MS volume may be explained by the method used to measure MS dimensions as previously explained. In PDC subjects, the canines are positioned in the palate and are close to the MS, therefore, their presence in the palatal area may have reduced MS volume in these subjects.

In both BDC and PDC displaced sides, the maxillary canine root tips were closer to the anterior MS wall than in the non-displaced sides. Because the MS length in this study was similar between the displaced and the non-displaced sides, this finding may be explained by the position of the displaced canine root and not to an increased MS length in the displaced sides.

It has been reported that the maxillary ectopic canines cause displacement of the midline toward the non-displaced side [24]. This midline displacement may affect the MS dimensions in the non-displaced side making it smaller. In the current study, MS width was similar between the displaced and non-displaced sides. However, the effect of the PDC displacement on the width of MS may have been masked by the great variability in the position of displaced canines in relation to MS wall and the possible increased transverse arch width in PDC side [24].

In the current study, significant differences between BDC and PDC were detected. In the displaced sides, BDCs were more laterally and more inferiorly located and the MS volume was increased. In the non-displaced side, PDCs were more laterally placed compared to BDCs which may suggest a larger maxillary width in PDC subjects. This was in agreement with Al-Nimri and Gharaibeh [25] who investigated arch width of Jordanian subjects with PDCs and reported that the transverse arch dimension was significantly wider in the PDC group. However, the increased lateral distance in the non-displaced side of the PDCs as measured from the maxillary canine crown tip to the MS wall was not associated with a wider MS or larger MS volume. This was in disagreement with AlHazmi [26] who reported a strong correlation between maxillary arch width and MS volume. Anyway, this distance (crown tip to MS) does not reflect the true maxillary arch width due to the large MS width variability in these subjects.

Regression analysis revealed that PDCs are associated with smaller MS volume, increased MS height and Class III skeletal pattern. This finding is in partial agreement with Oz et al. [13] who reported that impacted canines have smaller MS volume if the impacted canines were positioned high and closer to MS and that MS dimensional changes were associated with orthodontic traction of the impacted canines. In addition, regression analysis revealed that the OR of having PDC is reduced in a class II skeletal pattern. This finding was in agreement with Al Balbeesi et al. [27] who reported that the lowest frequency of canine impaction was found in patients with a Class II skeletal discrepancy and Basdra et al. [28] who observed impacted canines in 9% of Class III subjects compared to 1.3% in Class II subjects. On the other hand, this finding was in contrary to others who concluded that skeletal Class III subjects did not show a different prevalence of canine impaction [29] and that PDCs are not associated with altered skeletal features [30]. Furthermore, in the current study, maxillary canine displacement was not associated with the vertical skeletal relationship. This was in disagreement with the three times higher canine impaction reported in hypodivergent patients compared to normal face subjects [31].

Limitations of this study include a high female/male ratio and the number of subjects with a reduced vertical pattern was small. It has been suggested that subjects with a reduced vertical relationship have an increased maxillary sinus width and height compared to subjects with increased vertical skeletal pattern [15].

The findings of this study spot the lights into the importance of the assessment of MS during orthodontic treatment planning for patients with maxillary canine displacement. Early diagnosis and treatment of PDC could improve the MS volume and dimensions. In addition, clinicians would be aware of the challenges during orthodontic traction of maxillary displaced canines in presence of anterior MS pneumatization and improve orthodontic treatment outcome.

## Conclusions

- In PDC subjects, MS volume was reduced in displaced compared to non-displaced sides.
- In BDC subjects, MS volume was increased in displaced compared to non-displaced sides.
- In PDC and BDC subjects, the MS dimensions were similar between displaced and non-displaced sides.
- PDC subjects have a reduced MS volume and the canine crowns positioned closer to the MS wall laterally and vertically compared to BDC subjects.
- There was a statistically significant association between the presence/location of septa on the non-displaced side and the type of canine displacement.
- Anterior MS pneumatization was associated with the type of canine displacement.
- In BDC subjects, the MS was extended to the incisor region in 10% and to the canine region in 48% and 34% of the displaced and non-displaced sides, respectively.
- In PDC subjects, the MS was extended to the incisor region in 13% and 6% and to the canine region in 23% and 36% of the displaced and non-displaced sides, respectively.
- Palatal maxillary canine displacement showed negative association with the MS volume and positive association with the MS height.
- In subjects with Class III skeletal pattern, the odds of developing PDC increases by 11 times compared to Class II subjects.

## Data Availability

The data analyzed during the current study will be available from the corresponding author upon reasonable request.
