# Correction: Evaluation of maxillary sinus dimensions and volume using cone beam computed tomography in patients with unilaterally displaced palatal and buccal maxillary canines

**DOI:** 10.1007/s11282-023-00674-x

**Published:** 2023-02-25

**Authors:** Elham S. Abu Alhaija, Ahed M. AlWahadni, Akram Al-Tawachi, Saba O. Daher, Hasan O. Daher

**Affiliations:** 1 College of Dental Medicine, QU Health, Qatar University, P.O. Box: 2713 Doha, Qatar; 2 Department of Prosthodontics, Faculty of Dentistry, Jordan University of Science and Technology, Irbid, P.O. Box 3030 Jordan; 3 Private Practice, Dubai, United Arab Emirates; 4 Faculty of Medicine, Jordan University of Science and Technology, Irbid, P.O. Box 3030 Jordan

**Correction: Oral Radiology** 10.1007/s11282-022-00663-6

In the original publication of the article, the university name was missed in “Material and methods, CBCT images, and Declaration sections”.

The university name is given in this Correction

**Material and methods**

This study was reviewed and approved by the Research Ethical Committee (IRB)/Jordan University of Science and Technology (JUST). The sample of this study was collected over a period of 7 years by three means: database search (existing CBCT that were taken previously for diagnostic purposes as part of comprehensive orthodontic treatment), patients attending orthodontic clinics at the postgraduate dental clinics/Jordan University of Science and Technology (JUST) and referrals by fellow dentists and orthodontists. CBCT images were taken at the Dental Teaching Clinics (DTC)/Jordan University of Science and Technology (JUST).

**CBCT images**

A CS 9500 Cone Beam 3D System (Carestream Health, Ro-chester, NY, USA) with a flat panel detector located at the DTC/JUST was the only CBCT apparatus used.

**Declaration**

**Ethics approval** This study was reviewed and approved by the Research Ethical Committee (IRB)/Jordan University of Science and Technology (JUST).

The original article has been corrected.

